# Prediction and SHAP Analysis Integrating Morphological and Hemodynamic Parameters for Unruptured Intracranial Aneurysm Occlusion After Flow Diverter Treatment

**DOI:** 10.1111/cns.70386

**Published:** 2025-04-16

**Authors:** Hongchen Zhang, Chuanhao Lu, Zhen Hu, Deyu Sun, Liang Li, Hongxing Wu, Hua Lu, Bin Lv, Jun Wang, Shuhui Dai, Xia Li

**Affiliations:** ^1^ Department of Neurosurgery, Xijing Hospital The Fourth Military Medical University Xi'an China; ^2^ Institute for Health Informatics University of Minnesota Minneapolis Minnesota USA; ^3^ David Geffen School of Medicine University of California Los Angeles Los Angeles California USA; ^4^ Department of Neurosurgery People's Hospital of Xinjiang Uygur Autonomous Region Urumqi China; ^5^ Department of Neurosurgery The First Affiliated Hospital of Nanjing Medical University Nanjing China; ^6^ Department of Neurology, The First Medical Center Chinese People's Liberation Army General Hospital Beijing China; ^7^ Department of Neurosurgery, Xijing 986 Hospital The Fourth Military Medical University Xi'an China; ^8^ National Translational Science Center for Molecular Medicine and Department of Cell Biology The Fourth Military Medical University Xi'an China

**Keywords:** aneurysm occlusion, computational fluid dynamics, flow diverter, hemodynamic, intracranial aneurysm, morphology

## Abstract

**Background:**

Although most unruptured intracranial aneurysms (UIAs) have good prognosis after flow diverter (FD) treatment, some remain unoccluded for extended periods, posing a persistent rupture risk. This study aims to develop a predictive model for UIA occlusion after FD treatment through integrating morphological and hemodynamic parameters, which may be critical for personalized postoperative management.

**Methods:**

Data from patients with single UIAs treated with stand‐alone FD were collected from June 2018 to December 2022 in four cerebrovascular disease centers. Morphological parameters were obtained from 3D reconstructed aneurysm models, and hemodynamic parameters were derived by computational fluid dynamics (CFD) analysis. A predictive model for aneurysm occlusion was constructed using various machine learning algorithms, including logistic regression, Random Forest, XGBoost, and K‐Nearest Neighbors. Model performances were evaluated through repeated cross‐validation, 0.632 bootstrap, and 0.632+ bootstrap. Shapley additive explanation (SHAP) analysis was employed to assess the contribution of each parameter to UIA occlusion.

**Results:**

Seventy‐nine patients were reviewed; a total of 51 cases met the criteria, with an average age of 53.9 ± 9.9 years. The average aneurysm diameter was 3.72 ± 2.72 mm, comprising 29 occlusions and 22 non‐occlusions. Five variables were selected for further modeling, including follow‐up time > 6 months, aneurysm rupture ratio (ArR), occlusion ratio (OsR), parent artery wall shear stress (WSS), and the change of parent artery WSS. Logistic regression outperformed other algorithms, achieving an area under the curve (AUC) above 0.75, indicating good predictive performance. SHAP analysis revealed that the change of parent artery WSS contributed most significantly to accurate and early prediction. Additionally, a web application software was developed to assist clinicians in real‐time aneurysm occlusion prediction.

**Conclusions:**

This study developed a robust predictive model for UIA occlusion following FD treatment by integrating morphological and hemodynamic parameters, which may provide potentially valuable decision‐making support for optimizing treatment strategies.

## Graphical abstract

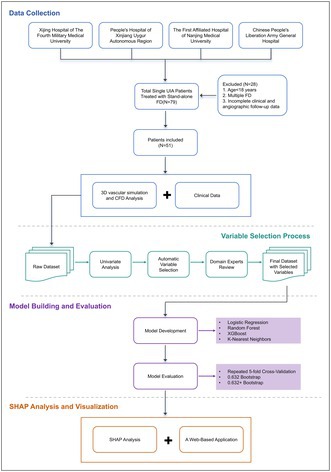

## 1 Introduction

Intravascular treatment has become the primary approach for managing unruptured intracranial aneurysms (UIAs) [1, 2]. The introduction of flow diverter (FD) technology has further advanced the concept of aneurysm intervention from “sac filling” to “vessel reconstruction” [3], significantly reducing the incidence of aneurysm retreatment [4]. Recently, the application of FDs has extended to include not only large but also gradually small and medium‐sized aneurysms [5, 6, 7, 8, 9]. However, some aneurysms remain incompletely occluded following FD implantation, posing a higher potential risk of delayed aneurysm rupture and significantly leading to poor prognosis [10, 11, 12]. Furthermore, due to the higher metal coverage, patients receiving FD treatment typically require dual antiplatelet duration (DAPT) for a longer duration compared to those with bare‐metal stents [13, 14]. DAPT is necessary to prevent thromboembolic complications after FD implantation. However, the duration of DAPT is often determined empirically by different centers. Prolonged DAPT may increase the risk of hemorrhagic complications, and some studies have also found that an abbreviated duration of DAPT lasting 6 months may be more appropriate to promote timely aneurysm occlusion [15, 16]. In light of these risks and contradictory clinical situations, precise assessment of aneurysm occlusion through appropriate follow‐up strategies is also crucial for determining whether to discontinue or continue DAPT therapy.

Several studies have identified the core factors for predicting UIA occlusion status after FD treatment, such as advanced age, smoking status, follow‐up duration, aneurysm diameter, aneurysm ostium ratio (OsR), and neck ratio (NR) [17, 18]. Additionally, significant hemodynamic changes occur in both the aneurysm and parent artery post‐FD, which can be quantified and visualized using computational fluid dynamics (CFD) methods [19]. Mut et al. found that parameters like inflow volume, inflow velocity, and wall shear stress (WSS) reduction post‐FD are closely related to aneurysm occlusion and are critical for determining the speed of aneurysm occlusion [20, 21, 22]. Furthermore, Cebral et al. discovered that parameters such as the inflow concentration index (ICI) and residual flow volume (RFV) correlated with the occlusion grade of aneurysms during follow‐up [23, 24]. These studies provide valuable insights into the post‐treatment healing status of aneurysms. However, most existing modeling methods focus exclusively on either morphological or hemodynamic parameters, respectively, without fully exploring the combined effects at the patient level. This limits the understanding of how the above variables dynamically contribute to the predictions, particularly in studies with small sample sizes.

Based on previous research, we naturally hypothesized that integrating morphological and hemodynamic parameters may be more effective in predicting UIA occlusion after FD treatment. In this study, we employed various machine learning algorithms, including logistic regression, Random Forest, XGBoost, and K‐Nearest Neighbors, to construct the predictive model. Model performances were evaluated through repeated cross‐validation, 0.632 bootstrap, and 0.632+ bootstrap. Subsequently, Shapley additive explanation (SHAP) analysis was then employed to quantify the contribution of individual factors to aneurysm occlusion [25]. Additionally, we developed a web application based on the aforementioned model to assist clinicians in real‐time aneurysm occlusion predictions. Overall, this study aims to integrate morphological and hemodynamic parameters to establish a predictive model for UIA occlusion post‐FD, effectively assess individual patient risks, and provide valuable personalized decision‐making for postoperative management.

## 2 Methods

**FIGURE 1:**
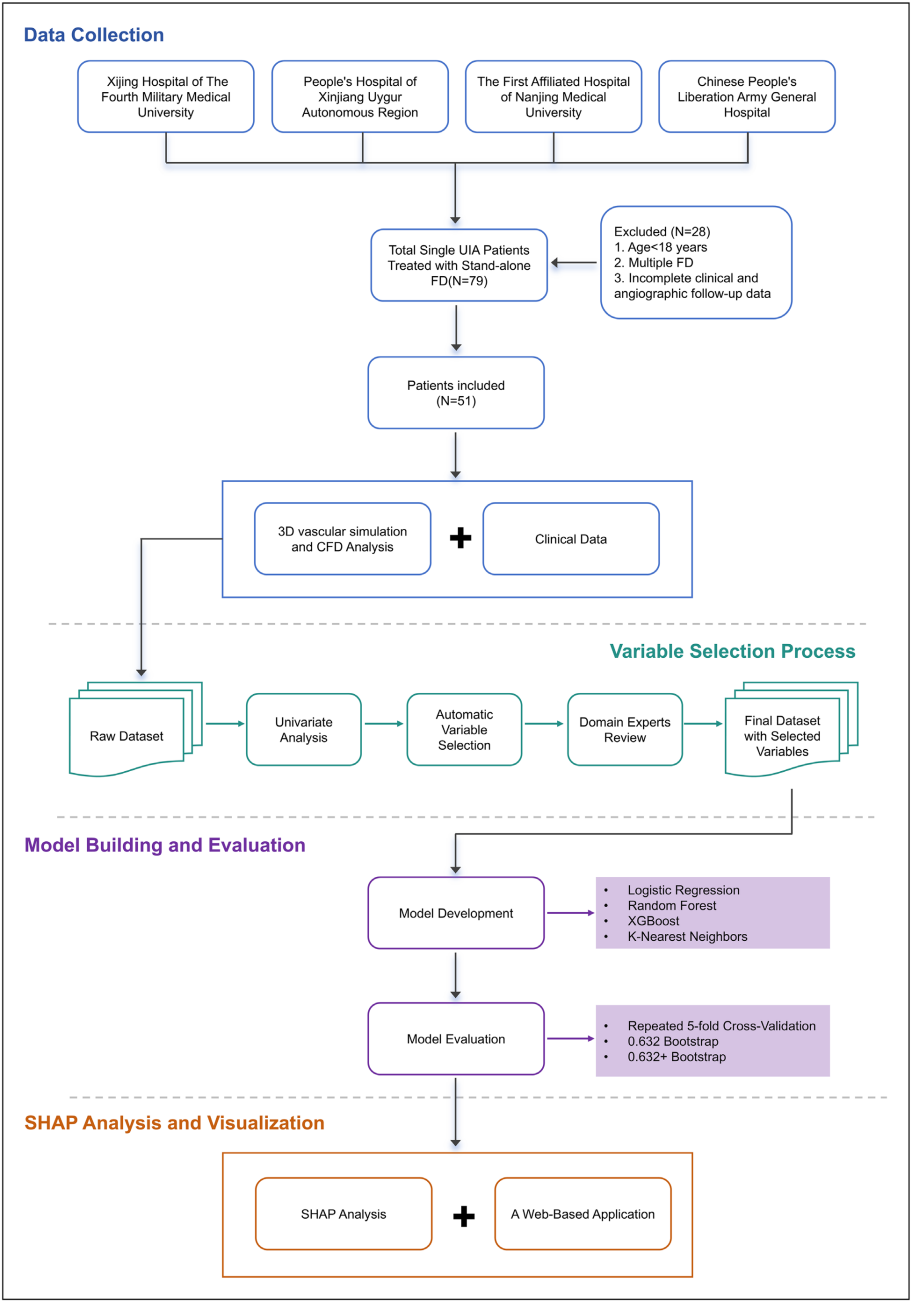
Flowchart for the predictive modeling of aneurysm occlusion post‐FD.

We developed predictive models for outcomes in UIAs following FD treatment (post‐FD) (Figure 1). The modeling was executed on a Python infrastructure (version = 3.11.5, USA).

### 2.1 Study Population and Clinical Data

This multicenter retrospective study included 79 patients with single UIA of the internal carotid artery treated with stand‐alone FD between June 2018 and December 2022, conducted across four major neurovascular disease centers located in the central, western, eastern, and northern regions of China: Xijing Hospital of The Fourth Military Medical University, People's Hospital of Xinjiang Uygur Autonomous Region, the First Affiliated Hospital of Nanjing Medical University, and the First Medical Center of Chinese People's Liberation Army General Hospital. Twenty‐eight patients were excluded, including 1 under the age of 18, 15 with multiple FD, and 12 with Incomplete clinical and/or angiographic follow‐up data. Ultimately, 51 patients were included for subsequent analysis. This study was approved by the Ethics Committee of the First Affiliated Hospital of Fourth Military Medical University as the lead institution (approval number: KY20232263‐C‐1), with a waiver of informed consent. It utilized existing medical records without involving participant identity or privacy, ensuring no risk or harm to participants. The study adheres to the principles of the Declaration of Helsinki and the STrengthening the Reporting of OBservational studies in Epidemiology (STROBE) guidelines where applicable.

All cases, including high‐risk small aneurysms with characteristics such as irregular morphology, family history, atherosclerosis, or intimal thickening, underwent the implantation of a single Pipeline Embolization Device (PED, Medtronic, USA) or Tubridge (MicroPort, China) FD [26, 27, 28, 29]. The selection of PED and Tubridge was based on the surgeon's expertise and analysis using Aneuguide software (ArteryFlow, China) [30]. Preoperative platelet function testing was performed, and patients with clopidogrel resistance were managed with ticagrelor. Patients received preoperative DAPT for 5 days and continued for 6 months postoperatively, transitioning to single antiplatelet therapy (aspirin) for up to 1 year.

Clinical data for all patients, including age, gender, hypertension, diabetes, smoking history, FD type, and follow‐up time (defined as the duration from FD implantation to angiographic follow‐up), were collected. DSA images were evaluated by three experienced neuro‐interventional physicians blinded to the study. Based on the O'Kelly–Marotta (OKM) score [31] from DSA, aneurysms were categorized into occlusion (OKM D grade) and non‐occlusion groups (OKM A‐C).

### 2.2 Aneurysm and FD Geometry

The patient‐specific aneurysm models were reconstructed from pre‐FD DSA images using a level‐set segmentation algorithm available in the Vascular Modeling Toolkit (VMTK, http://www.vmtk.org/) [32]. The raw geometries acquired were trimmed and smoothed in Geomagic Studio v. 12.0 (Research Triangle Park, USA) to generate stereolithography models for subsequent analysis. The FD models were mimicked in MATLAB (MathWorks, USA) based on the actual data of FD.

### 2.3 Finite Element Analysis (FEA) Modeling of FD Deployment

The mechanical procedure of implanting the FD into the aneurysm model was simulated using the FEA simulation strategy previously reported [33].

### 2.4 Hemodynamic Emulation

CFD emulations were performed for both pre‐ and post‐FD models. ANSYS ICEM CFD v. 16.2 (ANSYS Inc., USA) was employed to create computational grids for both the untreated and FD‐treated models. The mesh file thus generated was imported into ANSYS CFX v. 2019 (ANSYS Inc., USA). The flow‐governing Navier–Stokes equation for steady state was resolved by presupposing a rigid, non‐slip vessel wall, along with a homogeneous, incompressible, laminar, Newtonian fluid with a density of 1056 kg/m^3^ and a viscosity of 0.0035 kg/m·s [34]. The inlet flow rate was set at 4.6 mL/s for the internal carotid artery [35]. The outlet conditions were calculated according to Murray's law of flow distribution and the conservation of mass [36].

### 2.5 Morphological Parameters

To quantify the extent to which the defect envelopes the parent vessel circumferentially, Paliwal et al. [18] developed two morphological parameters, namely the OsR and NR.

$$\mathrm{OsR}=\frac{A_{\text{ostium}}}{A_{\text{vessel}}}$$

$$\mathrm{NR}=\frac{\mathrm{CND}}{\left(\frac{D_{1}+D_{2}}{2}\right)}$$

**FIGURE 2:**
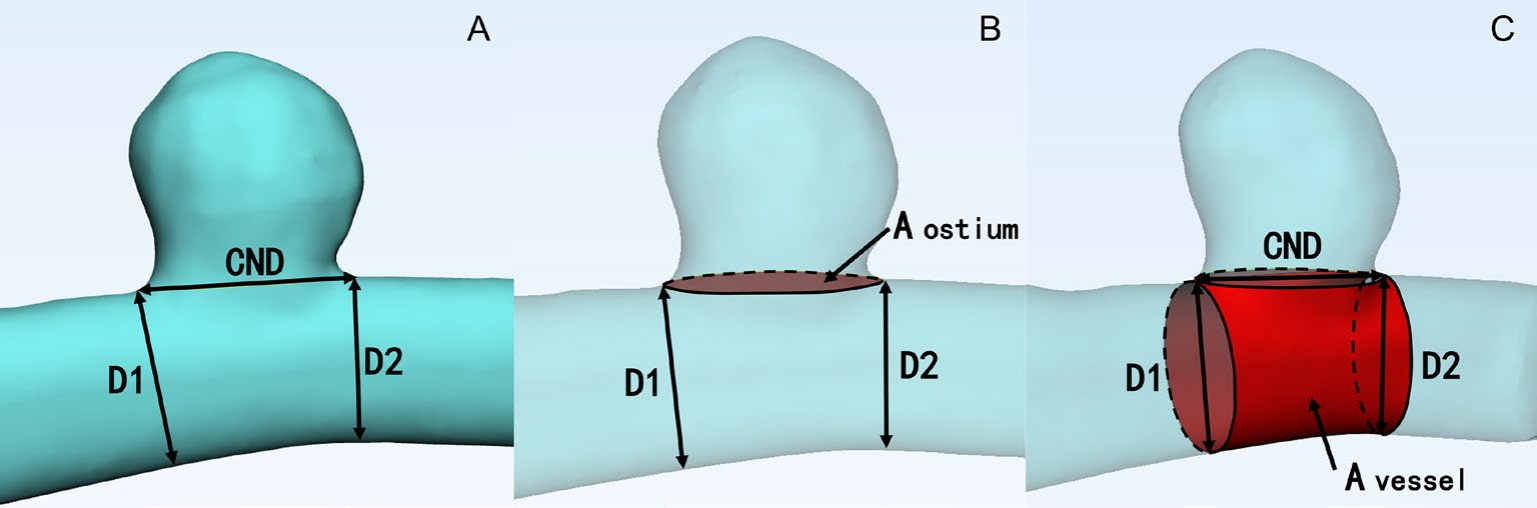
Definitions of OsR and NR on the 3‐D model. (A) CND and parent vessel diameters (D1, D2). (B) A_ostium_, the reconstructed area of aneurysmal ostium. (C) A_vessel_, the circumferential area of the remaining parent vessel.

The OsR is defined as the ratio of the reconstructed area of the aneurysmal ostium (A_ostium_) to the circumferential region of the remaining parent vessel (A_vessel_). The NR is defined as the ratio of the clinical neck diameter (CND) to the average of the vessel diameters measured at the proximal (D_1_) and distal ends (D_2_) of the aneurysmal neck. In our study, we have improved them to acquire more accurate values in well‐constructed 3D models (Figure 2).

We have also defined the area ratio (ArR) and volume ratio (VoR). ArR is defined as the ratio of the area of the reconstructed aneurysm surface (S_A_) to the circumferential area of the reconstructed artery (S_v_ = A_ostium_ + A_vessel_). VoR is defined as the ratio of the volume of the reconstructed aneurysm (V_aneurysm_: V_A_) to the volume of the reconstructed lesion artery (V_vessel_: V_v_).

$$A_{r}R=\frac{S_{A}}{A_{\text{ostium}}+A_{\text{vessel}}}$$

$$V_{o}R=\frac{V_{\text{aneurysm}}}{V_{\text{vessel}}}$$

### 2.6 Hemodynamic Parameters

Hemodynamic parameters were related to blood flow, flow rate, and WSS. Normalized WSS (NWSS) means that the aneurysm WSS is normalized by the parent artery WSS The normalized velocity is defined as the time‐averaged flow velocity within the aneurysm divided by the flow velocity in its parent artery. Ostium inflow area (OIA) is defined as the cycle‐averaged area of entering blood flow. RFV is defined as the volume of the flow in the aneurysm dome where the flow velocity exceeds a specified threshold [37]. ICI is defined as the degree of concentration of the flow entering the aneurysm [38].

### 2.7 Variable Selection

Patients' clinical data and parameters pre‐FD and changes in parameters post‐FD were collected as candidate variables. Descriptive statistics were calculated for both the occlusion and non‐occlusion groups. Univariate logistic regression was conducted to assess each variable's significance via *p*‐values. Lasso Regression was then utilized for the automated variable selection. Subsequently, experienced neurosurgeons reviewed the significant variables from both regression analyses, selecting five features for the model: follow‐up time, OsR, ArR, parent artery WSS (Pa), and the change of parent artery WSS (Pa). The variable “follow‐up time” was reassigned as “follow‐up time value.” If it exceeded 6 months, the value was set to 2.0.

### 2.8 Model Building and Evaluation

Multivariate logistic regression, Random Forest, Xgboost, and K‐Nearest Neighbors (KNN) were performed on the selected variables to develop a model to predict occlusion post‐FD therapy for UIAs. Given the limitation of a small sample size, three methods below were employed to evaluate the model performance from within.

### 2.9 Repeated 5‐Fold Cross‐Validation

This method improves the reliability of performance estimation by repeating the cross‐validation procedure multiple times. In our study, we employed fivefold cross‐validation and repeated it 10 times.

### 2.10 0.632 Bootstrap

The 0.632 Bootstrap variant assigns weights of 0.632 to in‐sample observations and 0.368 to out‐of‐sample observations. It is commonly used for bias correction in small sample sizes and addresses overfitting by providing a more realistic performance estimate.

### 2.11 0.632+ Bootstrap

The 0.632+ Bootstrap includes an adjustment factor to compensate for the training set's potential inadequacy in representing the population. This method refines bias correction by considering the frequency of observation appearances in bootstrap samples, providing an accurate estimate of the expected prediction error by mitigating overfitting and biases.

### 2.12 SHAP Analysis

After model training, we used SHAP to quantify each variable's contribution to the model's prediction. SHAP values, grounded in game theory's Shapley values, allocate “fair” importance scores by considering all possible feature combinations.

**FIGURE 3:**
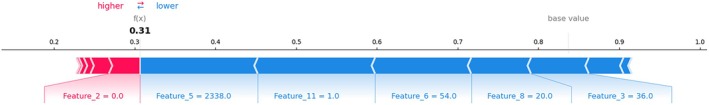
The force plot of the SHAP analysis.

Specifically, a force plot can illustrate each patient's case. For example, in the force plot (Figure 3), the bolded 0.31 represents the model's output score for this patient's outcome. A higher score prompts the model to predict 1, while a lower score prompts the model to predict 0. Variables are colored red and blue. Red signifies variables that elevate the model score, while blue signifies features that lower the model score. Variables with a more significant impact are closer to the red‐blue demarcation line (e.g., Features 2 and 5), with the bar size indicating the extent of the impact.

## 3 Results

### 3.1 Demographics and Characteristics of Aneurysms

**FIGURE 4:**
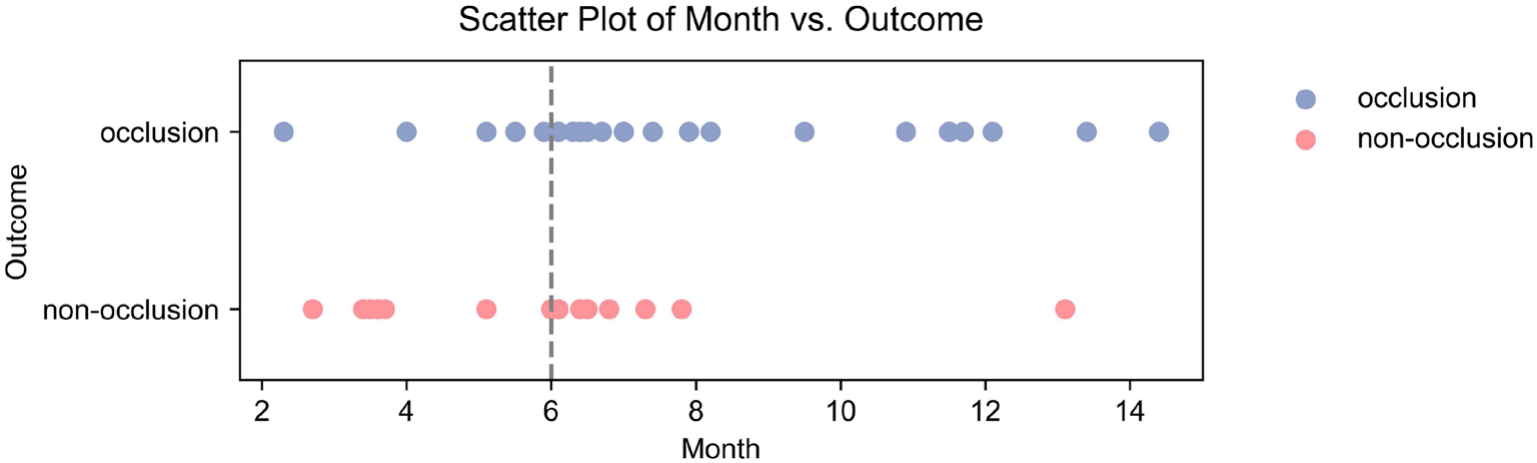
Distribution of follow‐up time and outcome.

This study included a total of 51 cases with an average patient age of 53.9 ± 9.9 years, comprising 13 males (25.5%) and 38 females (74.5%). There were 5 cases (9.8%) with a history of smoking, 19 cases (37.3%) with concomitant hypertension, and 4 cases (7.8%) with diabetes. The mean aneurysm diameter was 3.72 ± 2.72 mm. All patients successfully underwent FD implantation, with 37 PED (72.5%) and 14 Tubridge devices (27.5%) utilized. The average follow‐up time was 7.0 ± 3.0 months, with 15 cases followed for less than 6 months and 36 for 6 months or more (Figure 4). Among the cases, 29 aneurysms occluded (OKM Grade D), while 22 did not (OKM Grade A‐C) (Table 1).

**TABLE 1:** Baseline characteristics of UIAs.

Parameters	Average or frequency
Age (years)	53.9 ± 9.9
Female	38 (74.5%)
Smoking history	5 (9.8%)
Hypertension	19 (37.3%)
Diabetes	4 (7.8%)
Aneurysms size (mm)	3.72 ± 2.72
Type of FD	
PED	37 (72.5%)
Tubridge	14 (27.5%)
Follow‐up time (months)	7.0 ± 3.0
Follow‐up time ≥ 6 months	36 (70.6%)

*Note:* Data are shown as *n* (%) or the mean ± SD.

### 3.2 Descriptive Statistics, Univariate Analysis Results, and Variable Selection

Table 2 presents descriptive statistics for all variables across the two groups, including patient demographics, aneurysm morphological factors, and hemodynamic factors. Univariate logistic regression analysis indicated that the follow‐up time had a significant impact on aneurysm occlusion, while other patient clinical characteristics such as age, gender, history of hypertension, diabetes, smoking, and FD type did not. Among the morphological parameters, higher OsR and ArR contributed significantly to aneurysm occlusion, while no significant associations were observed for other morphological parameters (all *p* > 0.05). At the pre‐FD stage, no significant correlations were found between hemodynamic parameters and aneurysm occlusion (all *p* > 0.05). However, at the post‐FD stage, analysis of the changes in hemodynamic parameters from pre‐FD to immediate post‐FD indicated that the change of parent artery WSS significantly influenced the aneurysm occlusion.

**TABLE 2:** Results from univariate statistical analysis between the two groups.

Parameters	Occlusion group (*N* = 29)	Non‐occlusion group (*N* = 22)	*p*
Patient clinical characteristics			
Age	55.8 ± 8.8	52.4 ± 10.5	0.2186
Sex, female (%)	69.0	81.8	0.3016
Hypertension (%)	34.5	40.9	0.6386
Diabetes (%)	6.9	9.1	0.7735
Smoking (%)	13.8	4.5	0.2947
Type of FD, PED (%)	79.3	63.6	0.2188
**Follow‐up time**	8.1 ± 3.0	5.7 ± 2.3	**0.0090**
**Follow‐up time (> 6 months, %)**	79.3	59.1	**0.0150**
DAPT duration	6.2 ± 2.7	5.4 ± 2.2	0.2370
Aneurysm morphological parameters			
Size (mm)	3.894 ± 3.440	3.501 ± 1.308	0.6133
D1 (mm)	4.105 ± 0.6573	3.935 ± 0.7705	0.3937
D2 (mm)	3.909 ± 0.5098	3.678 ± 0.7154	0.1866
CND (mm)	8.210 ± 4.280	6.547 ± 4.187	0.1777
A_ostium_ (mm^2^)	9.589 ± 6.830	15.42 ± 18.07	0.1938
A_vessel_ (mm^2^)	106.1 ± 59.41	79.00 ± 73.86	0.1580
S_total_ (mm^2^)	160.2 ± 121.3	133.5 ± 102.6	0.4104
S_A_ (mm^2^)	54.10 ± 79.72	54.45 ± 41.30	0.9844
S_V_ (mm^2^)	115.7 ± 62.40	94.13 ± 77.71	0.2763
V_total_ (mm^3^)	190.5 ± 220.7	156.7 ± 149.8	0.5403
V_A_ (mm^3^)	64.94 ± 176.1	49.49 ± 54.84	0.6939
V_V_ (mm^3^)	125.6 ± 79.19	107.2 ± 105.9	0.4750
**OsR**	0.09631 ± 0.06618	0.2054 ± 0.1705	**0.0095**
NR	2.191 ± 1.214	1.708 ± 0.9082	0.1343
**ArR**	0.4431 ± 0.3157	0.6861 ± 0.3940	**0.0300**
VoR	0.4090 ± 0.6504	0.4822 ± 0.3307	0.6303
Hemodynamic characteristics			
Parent artery flow rate (mL/s)	4.327 ± 0.6076	4.424 ± 0.1597	0.4793
Aneurysm flow rate (mL/s)	1.019 ± 0.9066	1.198 ± 1.171	0.5358
Relative inflow	0.2462 ± 0.2265	0.2703 ± 0.2709	0.7254
OIA (mm^2^)	5.835 ± 4.687	7.576 ± 6.592	0.2825
ICI	0.5911 ± 0.5428	0.6702 ± 0.6861	0.6418
Aneurysm WSS (Pa)	4.575 ± 1.626	5.199 ± 1.897	0.2105
**Parent artery WSS (Pa)**	6.508 ± 2.386	7.876 ± 3.279	**0.0977**
Normalized WSS	0.7408 ± 0.2324	0.7220 ± 0.2713	0.7869
Area with WSS > 2 Pa (mm^2^)	36.13 ± 37.66	43.16 ± 30.89	0.4788
Proportion of area with WSS > 2 Pa (%)	78.72 ± 18.13	82.81 ± 18.40	0.4195
Aneurysm average flow rate (m/s)	0.2180 ± 0.07195	0.2227 ± 0.08027	0.8217
Parent artery average flow rate (m/s)	0.03684 ± 0.09153	0.04192 ± 0.01212	0.1005
Normalized flow rate	1.246 ± 3.496	0.5826 ± 0.2697	0.7319
RFV (mm^3^): V > 0.15 m/s	39.20 ± 113.0	30.56 ± 35.15	0.7295
Proportion of RFV: V > 0.15 m/s (%)	56.01 ± 19.74	62.34 ± 20.32	0.2618
Hemodynamic changes			
Aneurysm flow rate (mL/s)	−0.3816 ± 0.3924	−0.4547 ± 0.6696	0.6226
Relative inflow	−0.09561 ± 0.1099	−0.1029 ± 0.1555	0.8416
OIA	0.1049 ± 0.8372	0.5028 ± 1.382	0.2202
ICI	−0.2299 ± 0.2442	−0.2728 ± 0.3879	0.6278
Aneurysm WSS (Pa)	−2.526 ± 0.9779	−2.502 ± 1.299	0.9354
**Parent artery WSS (Pa)**	−3.456 ± 2.081	−2.039 ± 2.132	**0.0266**
Normalized WSS	0.04094 ± 0.3997	−0.08284 ± 0.3801	0.2670
Area with WSS > 2 Pa (mm^2^)	−20.74 ± 25.45	−22.87 ± 23.16	0.7547
Proportion of area with WSS > 2 Pa (%)	−43.29 ± 18.80	−40.07 ± 20.22	0.5564
Aneurysm average flow rate (m/s)	−0.1099 ± 0.04401	−0.1054 ± 0.06049	0.7625
Parent artery average flow rate (m/s)	0.01357 ± 0.01197	0.01488 ± 0.01322	0.7067
Normalized flow rate	−0.9601 ± 3.485	−0.2929 ± 0.1885	0.6361
RFV (mm^3^): V > 0.15 m/s	−27.75 ± 94.83	−18.29 ± 27.92	0.6569
Proportion of RFV: V > 0.15 m/s (%)	−32.52 ± 17.14	−35.01 ± 20.39	0.6239

*Note:* Data are shown as *n* (%) or the mean ± SD. S_total_ is the total surface area of the diseased vascular segment. V_total_ is the total volume of the diseased vascular segment. Significance of bold value indicates factors with *p* < 0.10, used for subsequent modeling analysis.

With a *p* < 0.10, OsR, follow‐up time, the change of parent artery WSS, ArR, and parent artery wall WSS were selected through univariate analysis. Lasso regression further identified ArR, OsR, the change of parent artery WSS, follow‐up time, and A_vessel_. After review by two experienced neurosurgeons, we ultimately selected five variables for further modeling: OsR, follow‐up time, the change of parent artery WSS, ArR, and parent artery wall WSS.

With a *p*‐value smaller than 0.10, OsR, follow‐up time value, the change of parent artery WSS, ArR, and parent artery wall WSS were selected through univariate logistic regression. Lasso regression selected ArR, OSR, the change of parent artery WSS, follow‐up time value, and A_vessel_. Based on the review of the above variable selection by two experienced neurosurgeons, we eventually selected five variables for further modeling: OsR, follow‐up time value, change of parent artery WSS, ArR, and parent artery wall WSS.

### 3.3 Results of Model Performance

**FIGURE 5:**
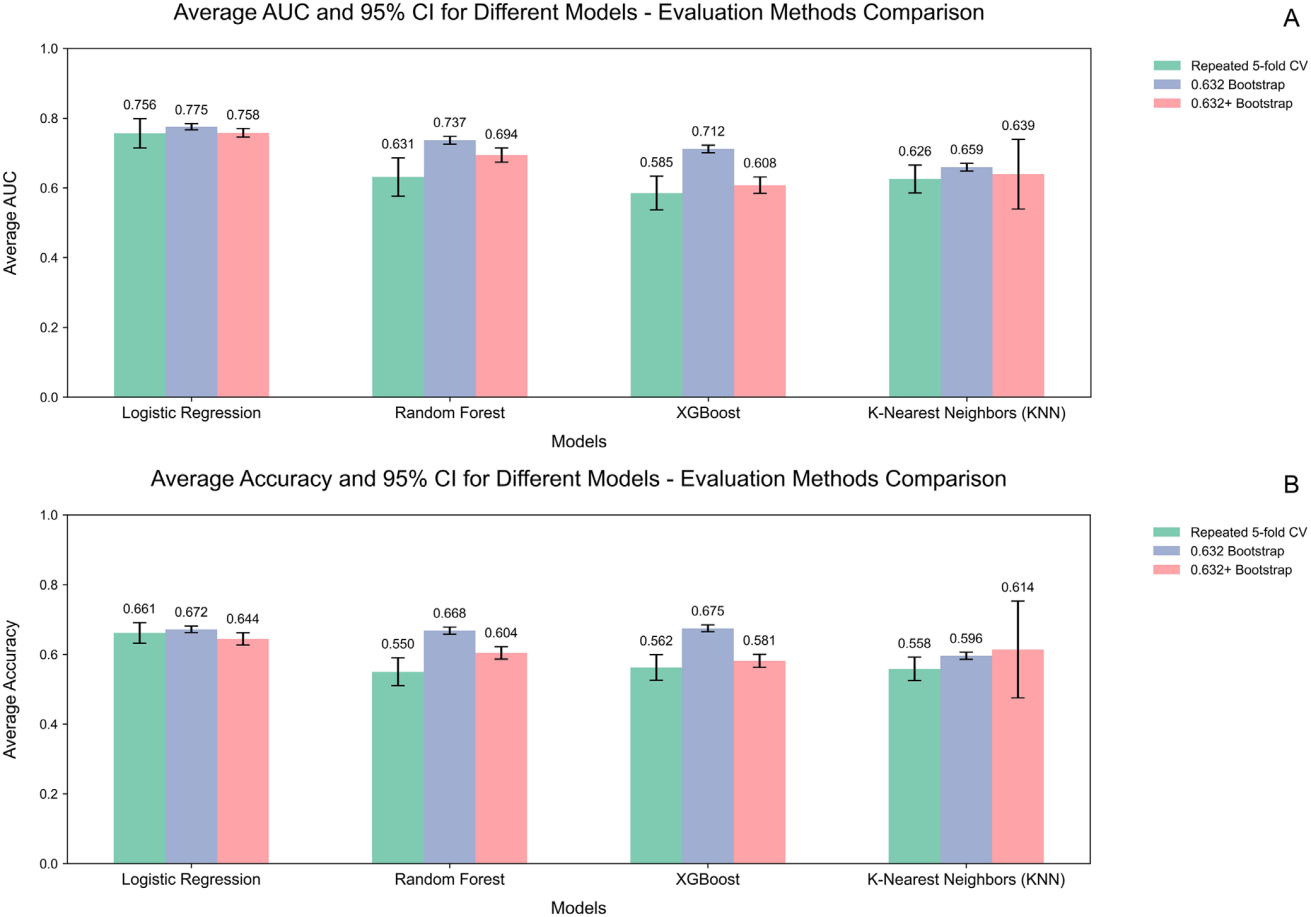
AUC and accuracy of different models.

As shown in Figure 5, multivariate logistic regression exhibits a decent model performance, consistently achieving an area under the curve (AUC) score exceeding 0.75 compared to the other three algorithms. In terms of accuracy, logistic regression consistently outperforms the other algorithms, with an average accuracy consistently exceeding 0.64. Typically, logistic regression is preferred for studies with small sample sizes due to its lower complexity and robust performance. Our results also confirmed this, illustrating its competitive performance compared to more complex algorithms.

### 3.4 SHAP Analysis

**FIGURE 6:**
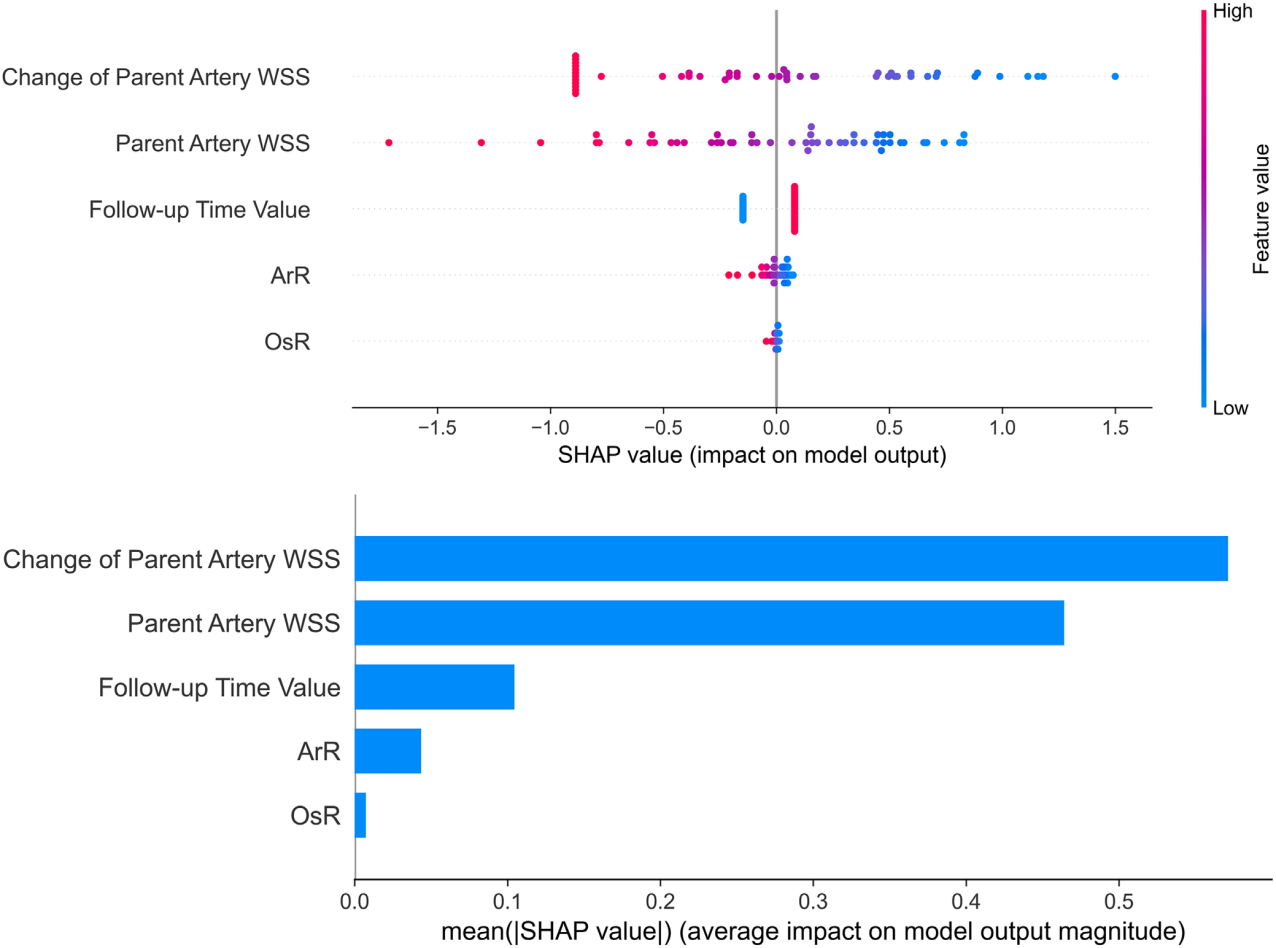
Visualization of SHAP analysis.

Figure 6 illustrates the five most significant features of the model. Each feature importance line displays the attributions of all patients, represented by differently colored dots; red dots indicate a higher likelihood of occlusion, while blue dots indicate a lower likelihood of non‐occlusion. Notably, as shown in Figure 4, the change of parent artery WSS emerges as the most influential feature for accurate predictions.

Figure 6 shows the five most significant features in the model. In each feature important line, the attributions of all patients to the results are plotted with differently colored dots, where red dots signify a higher possibility of occlusion and blue dots signify a lower possibility of non‐occlusion. As shown in Figure 6, the change in parent artery WSS stands out as the feature that contributes most to accurate prediction.

### 3.5 Typical Cases

Hemodynamic analysis and SHAP force plots for occluded and non‐occluded aneurysms are presented in Figures 7 and 8.

#### 3.5.1 Case of Occluded Aneurysms

**FIGURE 7:**
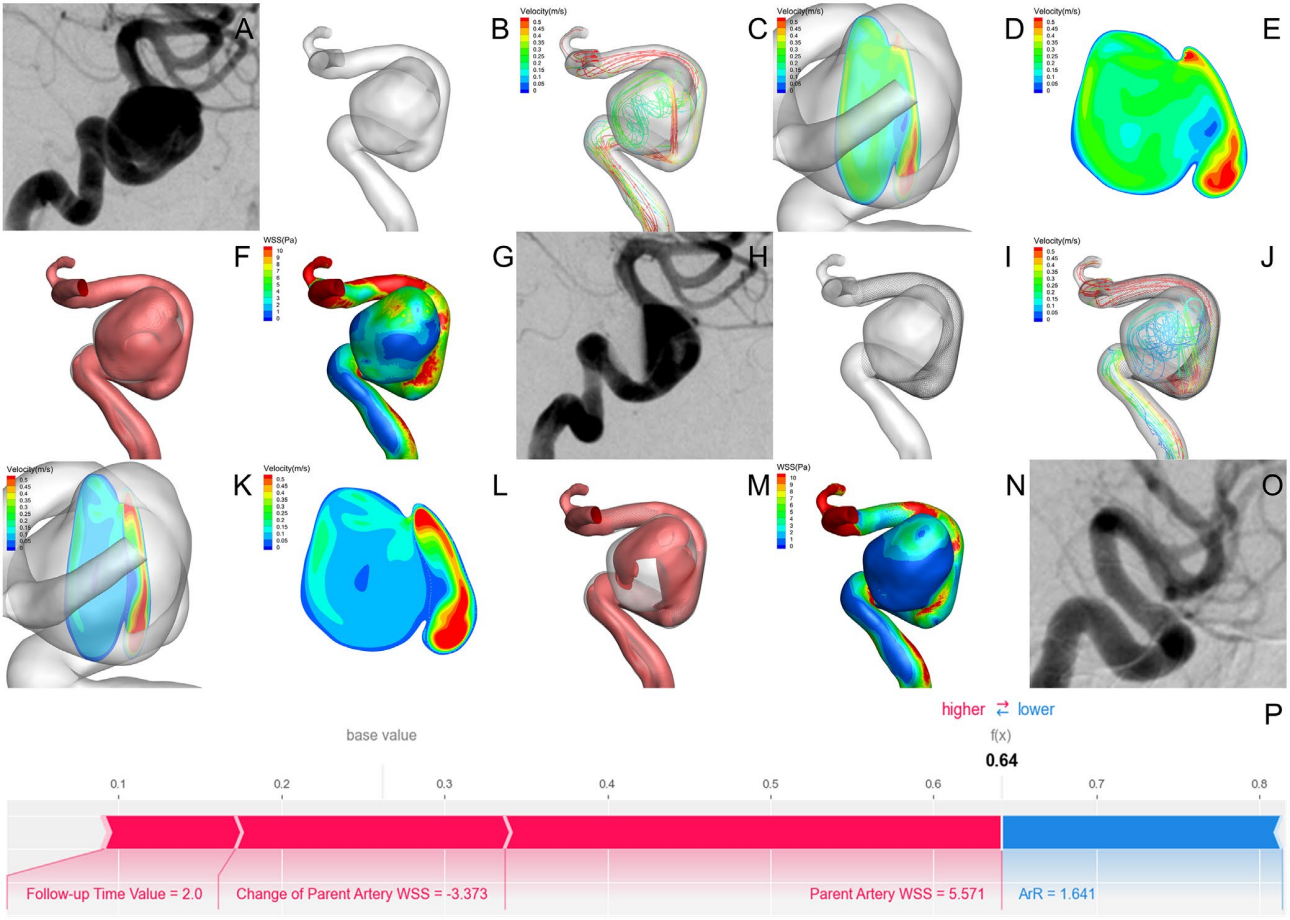
Case of occluded aneurysms. (A) Pre‐FD DSA image revealed a paraclinoid aneurysm. (B) 3‐D reconstruction model of the aneurysm and parent artery. (C–G) Results of pre‐FD hemodynamic simulation, including Velocity streamline map (C), Cross‐sectional cloud map of the aneurysm (D), Longitudinal cloud map of the aneurysm (E), High‐velocity blood flow region (v > 0.15 m/s), where the red portion represents high‐velocity blood flow (F), WSS distribution map (G). (H) Immediate post‐FD DSA image. (I) FEA modeling of FD implantation. (J–N) Hemodynamic changes immediately post‐FD, including: Velocity streamline map showed reduced blood flow velocity and decreased vortex (J), Cross‐sectional and longitudinal cloud map indicated decreased blood flow velocity in the aneurysm, with partial redirection to the parent artery (K and L), Reduction in the high‐velocity blood flow region (v > 0.15 m/s) (M), WSS distribution map showed decreased WSS in the aneurysm and parent artery (N). (O) 7.4‐month follow‐up post‐FD showed occlusion of the aneurysm. (P) The force plot of SHAP analysis, showing that the parent artery WSS (Pa) had the most substantial impact on elevating the score, while ArR contributed to a decrease in the score.

#### 3.5.2 Case of Unoccluded Aneurysms

**FIGURE 8:**
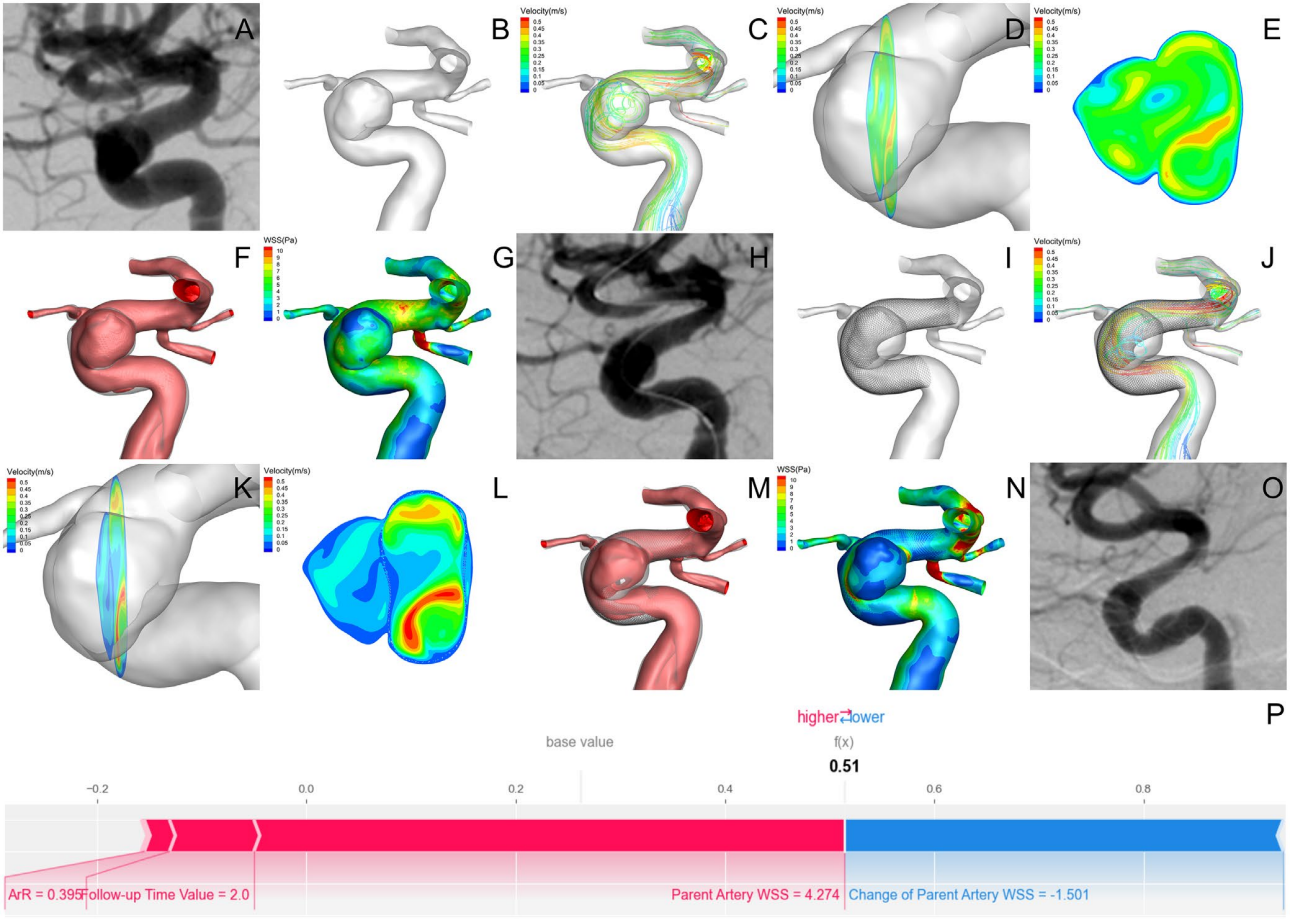
Case of unoccluded aneurysms. (A) Pre‐FD DSA image revealed a paraclinoid aneurysm. (B) 3‐D reconstruction model of the aneurysm and parent artery. (C–G) Results of pre‐FD hemodynamic simulation, including: Velocity streamline map (C), Cross‐sectional cloud map of the aneurysm (D), Longitudinal cloud map of the aneurysm (E), High‐velocity blood flow region (v > 0.15 m/s), where the red portion represents high‐velocity blood flow (F), WSS distribution map (G). (H) Immediate post‐FD DSA image. (I) FEA modeling of FD implantation. (J–N) Immediate post‐FD hemodynamic changes, including: Velocity streamline map showed reduced blood flow velocity and decreased vortex (J), Cross‐sectional and longitudinal cloud map indicated decreased blood flow velocity in the aneurysm, with partial redirection to the parent artery (K and L), Reduction in the high‐velocity blood flow region (v > 0.15 m/s) (M), WSS distribution map showed decreased WSS in the aneurysm and parent artery (N). (O) A 6.8‐month follow‐up post‐FD showed non‐occlusion of the aneurysm. (P) The force plot of SHAP analysis, showing that the parent artery WSS (Pa) was the primary variable that raises the score, while the change of parent artery WSS worked to lower the score.

### 3.6 A Web‐Based Application

**FIGURE 9:**
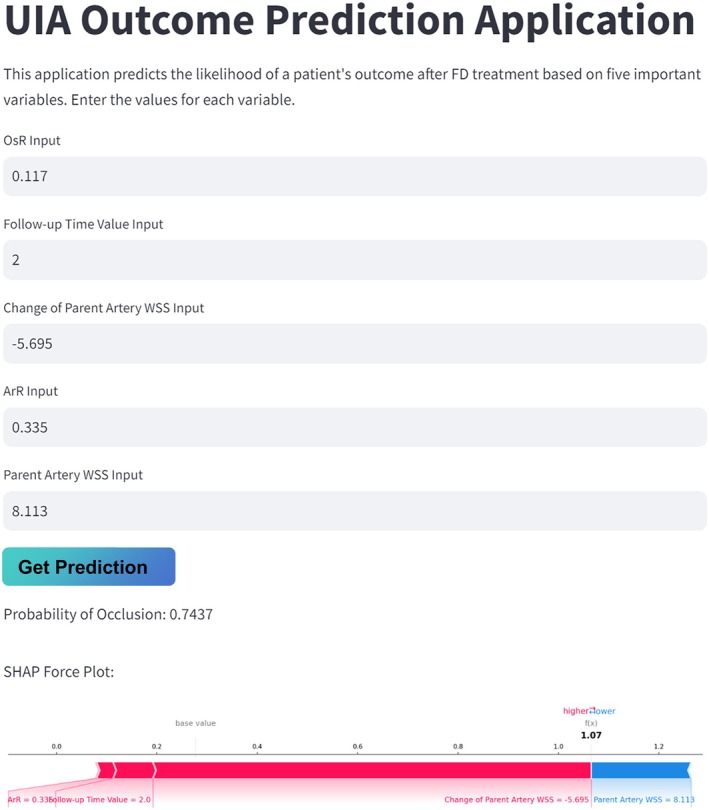
Application for UIA outcome prediction. This application predicts the likelihood of a patient's outcome after FD treatment based on five important variables. The values for each variable is entered. Then click the “Get Prediction” button, you will get the probability of UIA occlusion and the SHAP force plot.

We developed a web application where the user interface allows clinicians to input values for the five important variables. Once starting to process the values in the backend, the application interface instantly displays the predicted probability of the outcome (http://10.254.91.63:8501). Additionally, an individualized SHAP force plot is presented for further insights (Figure 9).

## 4 Discussion

Flow diverter promotes the gradual occlusion of aneurysms by reconstructing blood flow between the aneurysm and the parent artery [39]. However, some aneurysms still pose a potentially fatal risk of rupture and hemorrhage before complete occlusion, which may be associated with significant hemodynamic changes and prolonged antiplatelet drug therapy. Consequently, assessing whether the aneurysm is fully occluded is vital for guiding subsequent personalized treatment strategies. If occluded, preventive treatment for aneurysmal hemorrhage may be unnecessary; if not occluded, frequent angiography follow‐ups and anti‐hemorrhage treatment become essential. Therefore, it is crucial to accurately predict aneurysm outcome post‐FD. Based on logistic regression and SHAP analysis, we developed a model to predict UIA occlusion post‐FD integrating morphological and hemodynamic parameters. The model demonstrated good predictive performance (AUC > 0.75) using five key features including follow‐up time > 6 months, ArR, OsR, parent artery WSS, and the change of parent artery WSS. SHAP analysis allows clinicians to understand the contribution of each variable to UIA occlusion. In addition, we developed a user‐friendly web‐based application to assist clinicians in treatment decision‐making. This tool predicts aneurysm occlusion probabilities at various postoperative time points, which is critical for determining individualized follow‐up angiography schedules. This enables surgeons to accurately establish personalized follow‐up duration. Once the aneurysm is confirmed to be occluded, clinicians can focus more on preventing ischemic events, including in‐stent stenosis. Furthermore, results from personalized follow‐up angiographic assessments of in‐stent neointimal growth can also indirectly assist in guiding the adjustment of the DAPT regimen.

Numerous studies [17, 40] have shown a direct correlation between the UIA occlusion rate post‐FD and the duration from FD implantation to follow‐up. This study also indicated that the follow‐up time (*p* = 0.015) for the occlusion group was significantly shorter than for the non‐occlusion group. We incorporated follow‐up time as a key factor in developing a predictive model for UIA occlusion following FD treatment.

In recent years, morphological parameters integrating features of both aneurysm and parent artery have been found to predict aneurysm outcomes. Paliwal et al. [18] identified high OsR and NR as excellent indicators of incomplete occlusion post‐FD, possibly due to a larger proportion of the aneurysm ostium on the parent vessel leading to increased blood flow into the aneurysm sac. Another study also indicated that high OsR correlated with a higher intra‐aneurysmal flow rate and negatively correlated with 6‐month clinical outcomes [40]. In our study, OsR (*p* = 0.0095) was significantly higher in the non‐occlusion group, and SHAP analysis indicated that OsR was a crucial feature for predicting aneurysm occlusion. Additionally, studies by Fuga [41] and Kewlani et al. [42] showed that aneurysm volume was related to non‐occlusion following implantation of coils or Woven EndoBridge devices. We first studied the impact of the aneurysm's surface area and volume relative to the parent artery on occlusion and defined two additional morphological parameters, ArR and VoR, to quantify the extent of aneurysm expansion. The greater extent of aneurysm expansion enlarges the area of blood flow impact, further making thrombosis and occlusion more challenging. Our results confirmed that ArR (*p* = 0.030) has a potential predictive value for UIA occlusion after FD treatment.

Hemodynamic changes have been identified as crucial to the outcomes of UIAs [43]. Huang et al. [44] found significant reductions in aneurysm NWSS, inflow volume, and relative velocity, along with increased relative residence time after FD implantation in rabbit aneurysm models. The occluded group exhibited a higher relative residence time increment and percentage of inflow volume reduction. In the occluded group, the inflow stream was predominantly directed closer to the central region of the aneurysm neck, resulting in reduced turbulence within the aneurysm. This slower and simpler flow pattern is more conducive to aneurysm healing. Mut et al. [21] defined complete occlusion at 3 months or earlier post‐FD in clinical cases as “fast occlusion” and incomplete occlusion at 6 months as “slow occlusion.” They found that the slow occlusion group had significantly higher inflow rates, average flow velocities, and shear rates compared to the fast occlusion group. The above study suggested that the hemodynamic status post‐FD significantly influences the aneurysm occlusion rate [45]. In this study, univariate analysis found that the pre‐FD aneurysm WSS and parent artery WSS showed no significant associations with aneurysm occlusion (*p* > 0.05). However, the change of parent artery WSS post‐FD was more significant in the occlusion group, with a greater decrease (*p* = 0.0266), which slightly differed from the findings by Larrabide et al. [46] This discrepancy seems reasonable since the overall effects of FD may vary for different combinations of aneurysm and parent artery. Samaniego et al. [47] confirmed that parent arteries exhibit higher contrast enhancement in regions closer to the aneurysmal neck using a 7T high‐resolution MRI. This finding supported that a localized vasculopathy in the parent artery wall could lead to aneurysm formation and development. Therefore, predicting aneurysm occlusion through the change of parent artery WSS has certain clinical significance.

Nevertheless, we acknowledged the limitation of the small sample size. To address this, we employed fivefold cross‐validation, 0.632 bootstrap, and 0.632+ bootstrap to improve the reliability of performance estimation and mitigate the risk of overfitting in small sample models. We emphasized the necessity and challenges of future retrospective studies with larger sample sizes, which will further optimize the model's performance. Additionally, most aneurysms included in this study were small‐sized (diameter < 5 mm), with an average diameter of 3.72 mm. More and more physicians try to treat them with FD [7, 9, 48, 49]. We think our study may offer valuable insights into the postoperative management of small aneurysms. We also plan to extend the application of the predictive model to the occlusion of medium and large intracranial aneurysms and collect additional cases for multicenter validation.

## 5 Conclusions

We developed a robust predictive model for UIA occlusion post‐FD, integrating morphological and hemodynamic parameters. Logistic regression demonstrated good predictive performance using key features including follow‐up time > 6 months, ArR, OsR, parent artery WSS, and the change of parent artery WSS. SHAP analysis revealed that the change of parent artery WSS contributed most significantly to an accurate and early prediction. Additionally, we created a web‐based application to provide the predicted probability of occlusion and SHAP analysis results, which may assist clinicians in their treatment decision‐making. Future efforts should focus on refining these models to enhance clinical decision‐making and improve patient outcomes.

## Author Contributions

Conception and design: Xia Li, Shuhui Dai, and Hongchen Zhang. Acquisition of data: Hongchen Zhang, Chuanhao Lu, Liang Li, Hongxing Wu, Hua Lu, Bin Lv, and Jun Wang. Analysis and interpretation of data: Hongchen Zhang, Chuanhao Lu, and Zhen Hu. Drafting the article: Hongchen Zhang and Chuanhao Lu. Statistical analysis: Hongchen Zhang, Chuanhao Lu, Zhen Hu, and Deyu Sun. Critically revising the article: Xia Li, Hongchen Zhang, and Zhen Hu. Reviewed submitted version of manuscript: Xia Li and Hongchen Zhang. Approved the final version of the manuscript on behalf of all authors: Xia Li. Administrative/technical/material support: Zhen Hu and Deyu Sun. Study supervision: Xia Li and Shuhui Dai.

## Conflicts of Interest

The authors declare no conflicts of interest.

## Data Availability

All data can be obtained from the corresponding author, Xia Li (Email: lixia_fmmu@163.com) upon reasonable request.
